# Targeting Fluorescence Imaging of RGD-Modified Indocyanine Green Micelles on Gastric Cancer

**DOI:** 10.3389/fbioe.2020.575365

**Published:** 2020-09-25

**Authors:** Jun Shao, Xiaoming Zheng, Longbao Feng, Tianyun Lan, Dongbing Ding, Zikai Cai, Xudong Zhu, Rongpu Liang, Bo Wei

**Affiliations:** ^1^Department of Gastrointestinal Surgery, The Third Affiliated Hospital of Sun Yat-sen University, Guangzhou, China; ^2^Department of Biomedical Engineering, Ji’nan University, Guangzhou, China; ^3^Central Laboratory, The Third Affiliated Hospital of Sun Yat-sen University, Guangzhou, China

**Keywords:** gastric cancer, micelles, RGD, indocyanine green, targeting

## Abstract

Early diagnosis and complete resection of the tumor is an important way to improve the quality of life of patients with gastric cancer. In recent years, near-infrared (NIR) materials show great potential in fluorescence-based imaging of the tumors. To realize a satisfying intraoperative fluorescence tumor imaging, there are two pre-requirements. One is to obtain a stable agent with a relatively longer circulation time. The second is to make it good biocompatible and specific targeting to the tumor. Here, we developed an RGD-modified Distearyl acylphosphatidyl ethanolamine-polyethylene glycol micelle (DSPE-PEG-RGD) to encapsulate indocyanine green (ICG) for targeting fluorescence imaging of gastric cancer, aimed at realizing tumor-targeted accumulation and NIR imaging. ^1^H NMR spectroscopy confirmed its molecular structure. The characteristics and stability results indicated that the DSPE-PEG-RGD@ICG had a relatively uniform size of <200 nm and longer-term fluorescence stability. RGD peptides had a high affinity to integrin α_v_β_3_ and the specific targeting effect on SGC7901 was assessed by confocal microscopy *in vitro*. Additionally, the results of cytotoxicity and blood compatibility *in vitro* were consistent with the acute toxicity test *in vivo*, which revealed good biocompatibility. The biodistribution and tumor targeting image of DSPE-PEG-RGD@ICG were observed by an imaging system in tumor-bearing mice. DSPE-PEG-RGD@ICG demonstrated an improved accumulation in tumors and longer circulation time when compared with free ICG or DSPE-PEG@ICG. In all, DSPE-PEG-RGD@ICG demonstrated ideal properties for tumor target imaging, thus, providing a promising way for the detection and accurate resection of gastric cancer.

## Introduction

Gastric cancer (GC) is a common malignant tumor and ranks the second leading cause of cancer-related death in the world, making it a serious threat to human health and quality of life. According to GLOBACAN statistics, approximately 1,033,000 new cases are diagnosed and 783,000 deaths are estimated worldwide in 2018, the majority of which are diagnosed middle and advanced tumors (at stage II or III) (Bray et al., 2018; Son et al., 2019). Thus, accurate and early diagnosis of GC remains a significant clinical challenge. Recently, endoscopic procedures such as endoscopic mucosal resection (EMR) and endoscopic submucosal dissection (ESD), have been performed to treat GC in the early stage (Ban et al., 2018; Dahan et al., 2019). Meanwhile, laparoscopic gastrectomy has been widely accepted as a minimally invasive procedure for the treatment of GC (Zou et al., 2014; Itatani et al., 2019). Nevertheless, many questions remain unsolved. No or mild typical signs lead to great difficulty in the diagnosis of the early stage of GC. Also, due to the lack of tactile perception (haptic feedback), surgeons may have misjudgments about early lesions in laparoscopic surgery. The diagnosis of the small tumor, the accurate resection of the tumor, etc. Those seriously affect the quality of life and survival of patients with GC.

In the World Molecular Imaging Conference 2009, Roger Y. Tsien reported how to use the fluorescence microscope imaging system to guide the removal of fluorescence-modified tumor tissue in mice, which laid the foundation of the optical molecular imaging technology applied in the field of surgical navigation (Nguyen et al., 2010). Compared with traditional imaging techniques, intraoperative fluorescence imaging has several advantages, such as high contrast, high sensitivity, low cost, and visualization of tissues (Chen et al., 2017). To achieve a satisfying intraoperative fluorescence imaging, there are two prerequisites. One is to synthesize a stable agent with a relatively longer circulation time. The second is to make it good biocompatible and specific targeting to the tumor.

To date, many fluorescence imaging agents have been designed for tumor imaging and/or therapy, such as radionuclide, iron nanoparticles, and near-infrared (NIR) agents (Kotagiri et al., 2018; Lu et al., 2018). Among all the imaging agents, indocyanine green (ICG) is the only NIR agent approved for clinical use by Food and Drug Administration (FDA) (Hwang et al., 2017). It has been widely used for the determination of liver function and liver blood flow, cardiac output, and ophthalmic angiography (Hwang et al., 2017; Lee et al., 2019). However, there also exist several intrinsic drawbacks limiting the development of ICG, such as instability and self-aggregation in the liquid solution, resulting in fluorescence quenching, a short half-time in body, and a lack of tumor-targeted ability (Zhang et al., 2017). To address these challenges, numbers of carriers are introduced to encapsulate ICG that protect it from non-specific binding of plasma protein, provide enhanced stability and tumor targeting (i.e., permeability and retention effect, EPR). Nevertheless, in some solid tumors such as GC, since the interstitial fluid pressure is often increased (Yonucu et al., 2017; Hansem et al., 2019), it’s hard for these nanoparticles to permeate from leak tumor endothelial cells and largely transfer to the tumor sites. Therefore, challenges remain to improve the ability to penetration and targeting of the tumor.

Integrins, consisting of one α- and one β- submit, are a family of cell surface adhesion receptors that transmit bidirectional signals across the plasma membrane (Nieberler et al., 2017; Raab-Westphal et al., 2017). Now more and more evidence shows that integrins expressed by endothelial cells regulate cell migration and survival during angiogenesis, while integrins expressed by cancer cells enhance metastasis by promoting invasion and movement across blood vessels (Weis and Cheresh, 2011; Nieberler et al., 2017). Integrin α_v_β_3_ is over-expressed on neoplastic tumor blood vessels and some tumor cells, playing an important role in proliferation, metastasis, and invasion of cancer cells (Kwakwa and Sterling, 2017; Lin et al., 2018). A study on integrin α_v_β_3_ in GC cancer reveals that it is expressed widely in at least one tumor component and may be helpful in the routine classification of GC subtypes (Boger et al., 2015). The tripeptide Arg–Gly–Asp (RGD) can specifically bind to the integrin α_v_β_3_ and it is one of the most studied targeting molecules for cancer precision diagnosis and treatment. Several studies have shown that RGD linked to fluorescent molecules or radionuclides possess the function of tumor-targeted imaging, such as FITC, ^99m^ Tc, etc. (Zitzmann et al., 2002; Fang et al., 2011). However, because of the small molecular weight, such modified probes are too short-lived in circulation to be widely spread in clinical application.

**SCHEME 1:**
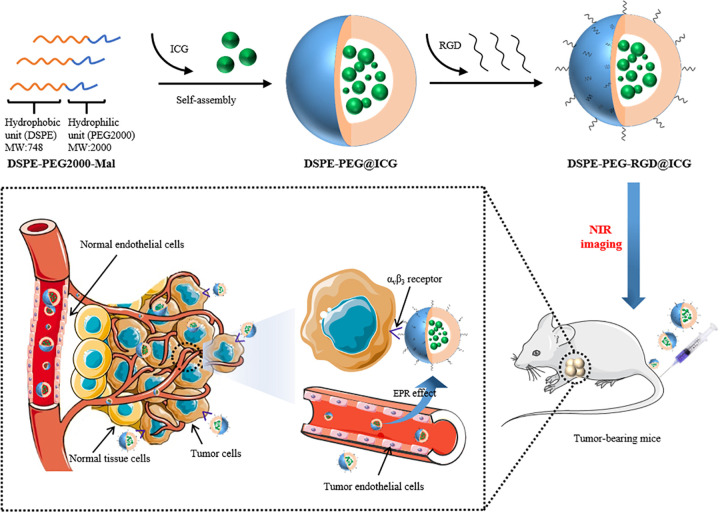
Schematic illustration of RGD targeted DSPE-PEG@ICG micelles for gastric cancer monitor.

Nowadays, nanocarriers, such as liposomes, polymer micelles, and nanoparticles, are widely applied in the drug delivery system (DDS) to achieve the maximum bioavailability and therapeutic effect (Repenko et al., 2017; Peng et al., 2018; Li et al., 2019). Lipid micelles are biocompatible, non-toxic, and stable *in vivo* (Xu et al., 2019). For example, PEGlyation has been largely used to modify the surface of nanocarriers for the purpose of stabilizing the DDS, weakening the interaction with plasma proteins, and reducing the clearance of reticuloendothelial system during the biological circulation (Gref et al., 2012). In this article, with the aim of tumor-targeted accumulation and NIR imaging, we designed and synthesized an RGD modified micelle to encapsulate ICG. As shown in Scheme 1, first, DSPE-PEG-RGD was obtained based on the Michael addition reaction between RGD peptide and DSPE-PEG-Mal. Second, the NIR agent ICG was encapsulated by the DSPE-PEG-RGD matrix via the self-assembly method. DSPE-PEG-Mal is an amphiphilic polymer. PEG serves as a hydrophilic unit to form the shell while DSPE is a hydrophobic unit to form the hydrophobic cavity with ICG in the core. The synthesis and Characteristics of DSPE-PEG-RGD@ICG were analyzed, including ^1^H NMR spectroscopy, transmission electron microscope (TEM), and so on. Then, the cellular uptake and biocompatibility were assessed. In addition, by using an optical imaging system, we observed the biodistribution and improved accumulation of DSPE-PEG-RGD@ICG in the tumor. The results of this study may provide a promising nanocarrier to realize the future clinical application of optical molecular imaging in early diagnosis and accurate resection of GC.

## Materials and Methods

### Materials and Reagents

Distearyl acylphosphatidyl ethanolamine – polyethylene glycol – maleimide (DSPE-PEG-Mal, Kw = 2,000) was bought from Shanghai Yare Biotechnology Co., Ltd. (Shanghai, China). ICG and dimethylsulfoxide (DMSO) were bought from Shanghai Macklin Biochemical Technology Co., Ltd. (Shanghai, China). RGD peptide was purchased from Shanghai Apeptide Co., Ltd. (Shanghai, China). Tetrahydrofuran (THF) was obtained from Damao Chemical Reagent Factory (Tianjin, China). RPMI 1640, cell culture reagents including Dulbecco’s modified Eagle’s medium (DMEM), fetal bovine serum (FBS), trypsin, and antibiotics were all bought from Gibco (Gaithersburg, MD, United States). Cell counting kit-8 (CCK-8) was obtained from Kaiji Bio-tech Co., Ltd. (Jiangsu, China). Human GC SGC7901 cells, mouse fibroblastic cells L929 were purchased from Beogene Biotechnology Co., Ltd. (Guangzhou, China). Live/Dead cell staining kits were obtained from BestBio Bio-Technology Co., Ltd. (Shanghai, China). All other reagents were of analytical grade unless otherwise noted.

### Synthesis of DSPE-PEG-RGD

Attachment of RGD peptide to DSPE-PEG was based on a reported method in the literature via the Michael addition reaction between RGD and DSPE-PEG-Mal, with a slight adjustment (Cureton et al., 2017). Generally, 20 mg of DSPE-PEG-Mal was dissolved in 5 ml of ultra-pure water and mixed with 6 mg RGD polypeptide. Then, gently stir the mixture for 4 h and followed by dialyzed in a dialysis bag (MWCO: 1,000 Da) for 24 h against ultra-pure water. The final product was obtained by freeze-drying and stored at −20°C.

### Synthesis of DSPE-PEG-RGD@ICG

DSPE-PEG-Mal was a long-chain polymer. PEG (MW: 2,000) served as a hydrophilic unit to form the shell while DSPE (MW: 748) was a hydrophobic unit to form the hydrophobic cavity with ICG in the core. DSPE-PEG-RGD@ICG was synthesized through the self-assembly method. In brief, 20 mg of DSPE-PEG-RGD and 6.7 mg of ICG were dissolved in 3 ml of THF and sonicated to ensure adequate dissolution and uniformity, followed by slowly adding 20 ml of ultra-pure water. Continuous sonicate for half an hour and dialyze the mixture in a dialysis bag (MWCO: 3,500 Da) for 24 h. The final product was obtained by freeze-drying and stored at −20°C. According to the same scheme, non-targeted DSPE-PEG@ICG was prepared by DSPE-PEG and ICG.

### Characteristics of DSPE-PEG-RGD@ICG

First, nuclear magnetic resonance (^1^H. NMR) spectrometer (AVANCE III 500M, Bruker, Germany) was performed to analyze the chemical structure of DSPE-PEG-RGD@ICG. Deuterated DMSO was used as a solvent. The morphology of DSPE-PEG@ICG and DSPE-PEG-RGD@ICG was illustrated by TEM (JEM-2010F, JEOL Ltd., Tokyo, Japan). The mean hydrodynamic diameter and size distribution of the micelles with or without RGD modified were measured by Zetasizer Nano-ZS90 (Malvern Instruments Ltd., Worcestershire, United Kingdom) at 25°C. Each sample was repeated three times for each measurement, and the average value was taken.

### Drug Loading Capacity and Encapsulation Efficiency

The encapsulation efficiency (EE) and drug loading capacity (LD) in both formulations were measured by UV-vis spectrometer (UV-3100PC, Mapada Instruments, Shanghai, China). Set the λ_max_ at 794 nm. The standard absorption curve of ICG in ultra-water was obtained and the formulas used were as follows:

$$\text{EE}\left(\%\right)=\frac{W\,e\,i\,g\,h\,t\,o\,f\,I\,C\,G\,i\,n\,m\,i\,c\,e\,l\,l\,e\,s}{W\,e\,i\,g\,h\,t\,o\,f\,f\,e\,e\,d\,I\,C\,G}\times 100\%$$

$$\text{LD}\left(\%\right)=\frac{W\,e\,i\,g\,h\,t\,o\,f\,I\,C\,G\,i\,n\,m\,i\,c\,e\,l\,l\,e\,s}{W\,e\,i\,g\,h\,t\,o\,f\,m\,i\,c\,e\,l\,l\,e\,s}\times 100\%$$

### *In vivo* Stability Evaluation

The absorption and fluorescence emission spectra were used to assess the stability of free ICG and DSPE-PEG-RGD@ICG in the liquid solution. Store the prepared samples in the dark at 4°C for 4 days. At the scheduled time points (0, 24, 48, 72, and 96 h), a UV-vis spectrometer was used to examine the absorption spectra and a fluorescence spectrometer (HR2000+, Ocean Optics) was used to analyze the fluorescence spectra.

### Cell Lines and Mice

Human GC SGC7901 cells, mouse fibroblastic cells L929 were used in this paper. SGC7901 cells were cultured in RPMI 1640 while L929 cells were cultured in DMEM at 37°C in a humidified atmosphere containing 5% CO_2_. All of the media were supplemented with 10% FBS, 1% penicillin, and 1% streptomycin. Male BALB/c (nu/nu) nude mice (6–8 weeks) were purchased from Beijing Virton Li Hua Experimental Animal Technology Co., Ltd. (Beijing, China). All mice experiments were approved by the Ethics Committee of the Institutional Animal Care and Use Subcommittee of the Third Hospital of Sun Yat-sen University. Before experimentation, all mice were raised for 1 week to acclimatize to the laboratory environment.

### Cellular Uptake

The internalization and distribution of cells were observed by a laser confocal fluorescence microscope. Briefly, SGC7901 cells were seeded in 35-mm cell petri dishes (Nest, 801002) and cultured 24 h for cell attachment. The cell density per dish was 5 × 10^4^. Next, replace the media with new serum-free media containing DSPE-PEG@ICG and DSPE-PEG-RGD@ICG, respectively (an equivalent ICG concentration: 1 mg/ml). After incubated for 4 and 12 h, the cells were washed with PBS and fixed with 4% paraformaldehyde. Afterward, the cells were stained with 10 μg/ml DAPI for 10 min and washed with PBS. Finally, a laser confocal fluorescence microscope (Zeiss LSM 710, Germany) was used for observing the binding and internalization of the micelles. The parameters were set at λ_ex_ 405 nm for the nuclei and λ_ex_ 633 nm for ICG.

For further quantitative analysis, 1 × 10^5^ SGC7901 cells were seeded in 12-well plates per well and cultured with serum-free media containing DSPE-PEG@ICG and DSPE-PEG-RGD@ICG (an equivalent ICG concentration: 1 mg/ml) for 12 h. At predetermined times, the cells were harvested and washed with PBS. Then, the cells were re-suspended in PBS and immediately quantitatively analyzed by Accuri flow cytometry (BD, United States).

### *In vitro* Cytotoxicity

For the cell viability assessment, Cell Counting Kit (CCK-8) assay and live/dead staining assay were performed. Generally, SGC7901 and L929 cells in the logarithmic phase were harvested, resuspended, and seeded into 96-well at the density of 5 × 10^3^/ml. The cells were incubated overnight for adherents and replaced the media with new media containing the micelles with or without RGD peptide modified at different concentrations, respectively. Continue to incubate the cells for 24 h and assess the cell viability by CCK-8 assay. The absorbance value was measured at λ = 490 nm. Cell death was detected by the live/dead staining assay. In brief, SGC7901 cells were cultured with micelles with or without RGD peptide modified for 24 h, then stained with a mixed solution of calcein-AM and PI and observed under the fluorescence microscope. The images were acquired at 490 nm for calcein-AM and 545 nm for PI, respectively.

### *In vitro* Blood Compatibility Test

This section includes two parts: hemolysis assay of erythrocytes and blood clotting analysis (Huang et al., 2016). For hemolysis assay (Skalickova et al., 2017), the fresh blood sample was collected from a 6-week-old nude mouse and red blood cells (RBCs) were obtained by centrifuged at 5,000 rpm for 5 min, followed by purified and re-suspended in PBS. The final concentration of the RBCs solution was adjusted to 16% (v:v) diluted with PBS. Then, DSPE-PEG-RGD@ICG samples at different concentrations (0.1, 0.5, and 1.0 mg/ml) in 1 ml PBS were added to the RBCs solutions (50 μl) and incubated for a period of time (0.25, 0.5, 1, 2, 3, 6, 12, and 24 h). RBCs incubated with ultra-pure water and PBS solution with the same volume were respectively set as negative and positive controls. At the end of the experiment, centrifuged the mixed solutions at 1,000 rpm for 5 min and detected the absorbance of the supernatants containing lysed erythrocytes at 540 nm, which stood for the effect of DSPE-PEG-RGD@ICG on the dissolution of erythrocytes.

Next, activated partial thromboplastin time (APTT) and prothrombin time (PT) were adopted to assess the effect of DSPE-PEG-RGD@ICG micelles on the coagulation system (Pyataev et al., 2019). Briefly, fresh blood samples were collected as mentioned above and stabilized with sodium citrate. Centrifuged at 1,000 g for 10 min and the plasma was obtained. A total of 360 μl plasma was collected and mixed with 40 μl DSPE-PEG-RGD@ICG PBS solution (concentration: 0.1, 0.2, 0.5, and 1 mg/ml). Meanwhile, the same volume of plasma mixed with normal saline was set as a negative control. Added the detection reagents and analyzed the APTT and PT utilizing an automatic coagulation analyzer (STAR Evolution, Diagnostica Stago, Assiernes, France).

### *In vivo* Acute Toxicity Test

An acute toxicity test was performed for safety assessment *in vivo* (Paradossi et al., 2019). Twelve healthy male nude mice were allocated to four groups (*n* = 3) randomly and intravenously injected with DSPE-PEG-RGD@ICG at different does (an equivalent ICG dose: 0, 1, 2, and 4 mg/kg), respectively. Continue feeding the mice for one week and observe the death, diet, coat color, activity, and other conditions of the mice. Harvest blood samples via the retro-orbital vein on the 8th day and then kill the mice. The main organs were extracted immediately for sectioning and hematoxylin-eosin (H&E) staining. Histopathological analysis was conducted to assess organ toxicity. Blood samples were kept at 2–8°C. Use whole blood for cell count (RBC, WBC, and blood platelet) and serum for liver (ALT and AST) and kidney (Cr) function test to evaluate the effect of DSPE-PEG-RGD@ICG on bone marrow hematopoietic system and liver and kidney function in nude mice.

### *In vivo* Fluorescence Imaging and Distribution

To develop the tumor-bearing mice model, 5 × 10^6^ SGC7901 cells in PBS (0.2 ml) were injected into the left armpit subcutaneous areas of the male nude mice. The size of tumors was measured twice or three times a week and the volume was calculated (*V* = length × (width)^2^/2). When the tumor size reached ≈150–200 mm^3^, allocate the mice to three groups (*n* = 3) randomly, followed by treated with DSPE-PEG-RGD@ICG, DSPE-PEG@ICG or free ICG via tail vein (an equivalent ICG dose: 2 mg/kg), respectively. Mice were anesthetized with a mixture of 2% isoflurane and air. At selected time points of 0, 1, 3, 6, 24, and 48 h post-injection, the fluorescence signals were acquired with *Bruker in vivo Xtreme* imaging system (Billerica, MA, United States) with λ_ex_ = 730 nm and λ_em_ = 830 nm. After 48h imaging, all the experimental mice were immediately killed for isolating and visualizing tumors and main organs under the same conditions as described above. Last, *Bruker* molecular imaging software was used to quantify the fluorescence intensity at selected ROIs.

### Statistical Analysis

In this study, quantitative data was demonstrated as mean ± SD, and the statistical analysis was conducted utilizing SPSS 21.0 software (Chicago, IL, United States). *T*-test was applied for comparisons between two groups whereas a one-way analysis of variance (ANOVA) was used for comparisons among multiple groups. The differences were considered significant when ^∗^*P* < 0.05, and very significant when ^∗∗^*P* < 0.01.

## Results and Discussion

### Synthesis and Characterization of the DSPE-PEG-RGD@ICG

The preparation process of DSPE-PEG-RGD@ICG was shown in Scheme 1 and its molecular structure was further verified by ^1^H-NMR spectroscopy. As shown in Supplementary Figure S1, resonance peak at ∼3.4 ppm corresponded to δa (–CH_2_O–) of PEG, and resonance peak at ∼3.5 ppm represented δb (–OCH_2_–CH_2_–), both of which were unaffected by the reaction with RGD peptide. Resonance peak at ∼7.0 ppm corresponded to δc (–CO–CH =) of the Mal group, which nearly disappeared in the spectrum of DSPE-PEG-RGD. The residual resonance peak might be due to the residual impurities in the sample. Thus, the Mal groups of DSPE-PEG-Mal had successfully reacted with the thiol groups of RGD peptide (Mozhi et al., 2020).

**FIGURE 1:**
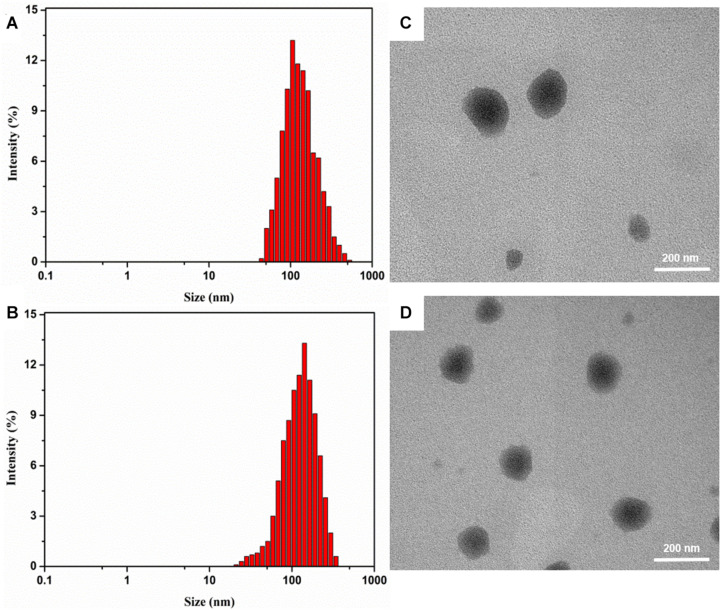
The size distribution and TEM result of DSPE-PEG@ICG **(A, B)** and DSPE-PEG-RGD@ICG **(C, D)**.

We further characterized the obtained micelles. The average size and surface potential were characterized by dynamic light scattering (DLS). The mean size of DSPE-PEG@ICG and DSPE-PEG-RGD@ICG were respectively 133.2 ± 4.2 nm and 147.6 ± 3.9 nm with a polydispersity index (PDI) of 0.21 and 0.28, as shown in Figures 1A,B, indicating slightly changes after the conjugation with RGD. Previous studies had revealed that particles with a <200 nm size increased drug accumulation ability in solid tumors through the EPR effect (Gao et al., 2009; Du et al., 2017). Meanwhile, the TEM image (Figures 1C,D) revealed that the morphology of the micelles was approximately circular and homogeneously distributed. The results were similar to that of DLS. The standard absorption curve of ICG in ultra-pure water was shown in Supplementary Figure S2. The LD and EE are crucial properties in drug delivery. In our study, the LD and EE of ICG were 11.4 and 34.2%, which were lower compared with previous studies (Xu et al., 2018). Similar results of micelles with or without RGD modifications were obtained.

### Spectral Properties and Stability

**FIGURE 2:**
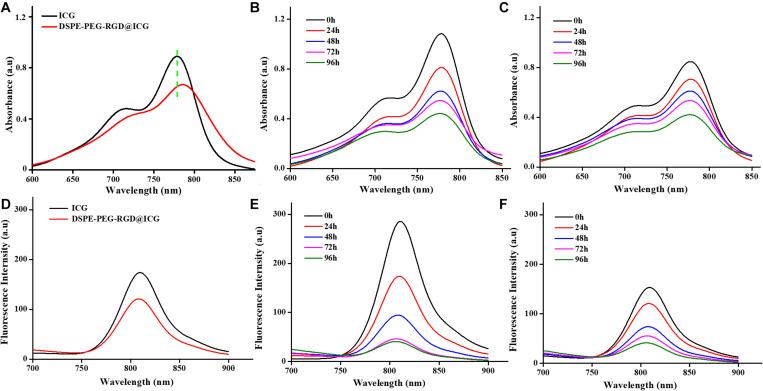
UV–vis absorption spectra **(A)** and fluorescence emission spectra **(D)** of free ICG and DSPE-PEG-RGD@ICG. Stability assessment of free ICG **(B, E)** and DSPE-PEG-RGD@ICG **(C, F)** for 4 days.

The spectral properties of ICG and DSPE-PEG-RGD@ICG were showed in Figures 2A,D. The UV absorption and FL emission of DSPE-PEG-RGD@ICG were similar to that of ICG dissolved in pure water. In detail, the peak absorbance of DSPE-PEG-RGD@ICG had a slight red shift (≈7 nm), which verified that ICG had been encapsulated into the micelles successfully. Moreover, a shift toward longer wavelengths would lead to a remarkable decrease in the background signal during detection, making it an improved signal-to-noise ratio *in vivo* (Proulx et al., 2010).

Previous studies have demonstrated that ICG is unstable in aqueous solutions due to the saturation of the double bonds in the conjugated chain. When the concentration of ICG exceeds 3.9 mg/ml, it aggregates to form dimers and oligomers, leading to fluorescence self-quenching and simultaneous decrease of fluorescence and absorption spectrum (Saxena et al., 2003). After reserved in the dark at 4°C for 4 days, the absorption and fluorescence spectrum of free ICG decreased by 60 and 90%, respectively (Figures 2B,E). On the contrary, the absorption and fluorescence spectrum of DSPE-PEG-RGD@ICG declined by 50 and 75%, respectively (Figures 2C,F). Obviously, when ICG was encapsulated in DSPE-PEG-RGD@ICG, the hydrophobic lipid end could prevent their aggregation, thus improving the stability of ICG in aqueous solution. In addition, previous studies have shown that the interaction between lipids and ICG can improve the NIR imaging performance of ICG and make it have deeper tissue penetration (Kraft and Ho, 2014). At the same time, in order to achieve effective tumor imaging and accumulation, it is necessary to maintain the fluorescence stability of ICG.

### *In vitro* Cellular Uptake

**FIGURE 3:**
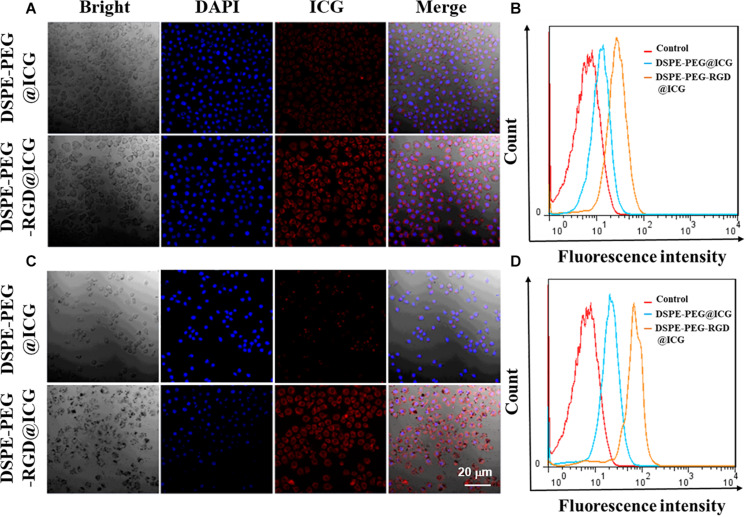
Confocal images and flow cytometry analysis of DSPE-PEG@ICG or DSPE-PEG-RGD@ICG by SGC7901 cells at 4 h **(A, B)** and 12 h **(C, D)**, respectively.

Integrin α_v_β_3_ is widely expressed and maybe a putative prognostic biomarker in GC tissues according to a large-scale study conducted by Boger et al. (2015). Human GC SGC7901 cells were also overexpressed integrin α_v_β_3_ (Cheng et al., 2017). In this section, in order to investigate the targeting ability of micelles with or without RGD, SGC7901 cells were incubated with micelles with or without RGD peptide modified for 4 and 12 h, respectively. Confocal microscopy was employed to observe the precise internalization procession. Nuclei represented blue fluorescence and ICG exhibited red fluorescence (Figures 3A,C). After incubated with DSPE-PEG-RGD@ICG for 4 or 12 h, the red fluorescence signal from the SGC7901 cells was significantly stronger than the cells incubated with DSPE-PEG@ICG, indicating that DSPE-PEG-RGD@ICG were greatly internalized by SGC7901 cells when compared to DSPE-PEG@ICG. Further flow cytometry analysis (Figures 3B,D) shown that the fluorescence intensity for DSPE-PEG-RGD@ICG in SGC7901 cells was significantly higher than that for DSPE-PEG@ICG at 4 or 12 h. Given the fact that RGD peptides have a strong affinity to integrin α_v_β_3_ and specifically target tumors, the attachment of RGD to micelles enhanced the cellular uptake. Additionally, previous researches have shown that cellular internalization largely relied on the reaction between RGD and its receptors on the cell surface. Wang et al. designed iRGD or RGD modified nanoparticles with enhancing tumor accumulation and penetration (Wang et al., 2014). Yan et al. (2016) demonstrated a higher cellular uptake of iRGD modified nanoparticles in breast cancer cells with higher expression level of integrin α_v_β_3_. The higher expression level of integrin α_v_β_3_, the more RGD modified nanocarriers tend to be phagocytosed.

### *In vitro* Cytotoxicity

**FIGURE 4:**
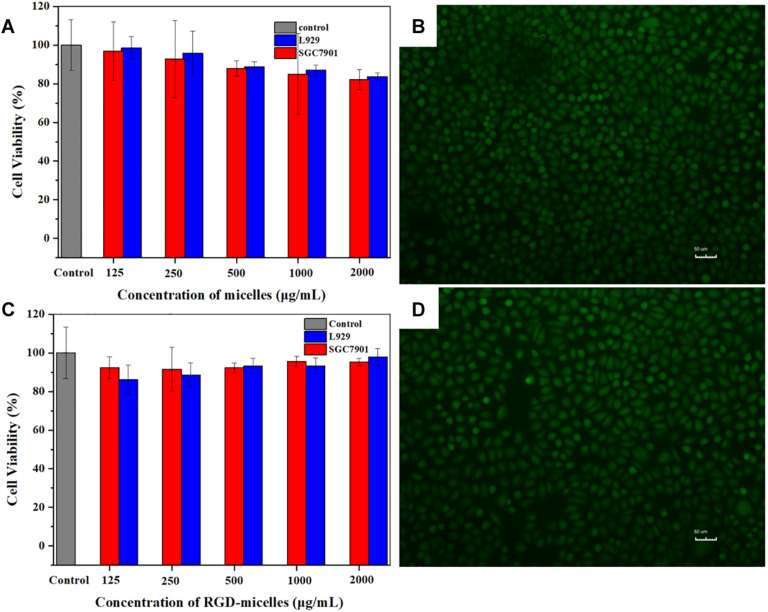
CCK-8 analysis of the cell viability after incubated with **(A)** DSPE-PEG@ICG or **(C)** DSPE-PEG-RGD@ICG for 24 h. Fluorescence images of SGC7901 cells cultured with **(B)** DSPE-PEG@ICG or **(D)** DSPE-PEG-RGD@ICG after Live/Dead staining.

The prerequisite property for a well-designed agent for clinical application is fine biological compatibility. To evaluate the biocompatibility of micelles *in vitro*, SGC7901 cells and L929 cells were cultured with the micelles with or without RGD peptide modified respectively. The relative growth rates (RGR) of cells were obtained and demonstrated in Figures 4A,C. With the increase in the concentration of micelles, there was a slightly correlated influence on the cell viability with no significant difference (*P* > 0.05), indicating no obvious toxicity. Moreover, even when the concentration increased to 2,000 μg/ml, cell viability was still more than 80%. The results of Live/Dead staining were shown in Figures 4B,D. After 24 h, there were a great number of viable cells and little dead or later apoptosis cells no matter incubated with DSPE-PEG@ICG or DSPE-PEG-RGD@ICG. The results were consistent with the CCK-8 assay. Based on RGR Score and Grade standard, once the material is considered to be utilized in medical applications, the RGR should ≥75%, which implies low or no cytotoxicity (Xiao et al., 2019). PEGylated materials are considered to be promising drug carriers with low cytotoxicity, biodegradability, and biocompatibility (Allen and Cullis, 2004). Meanwhile, ICG is approved for clinical use by FDA, and RGD peptides are naturally present in a variety of extracellular matrices. The expected results indicated that even a high dose of micelles could be applied to cells with little cytotoxicity *in vitro*.

### *In vitro* Blood Compatibility Test

**FIGURE 5:**
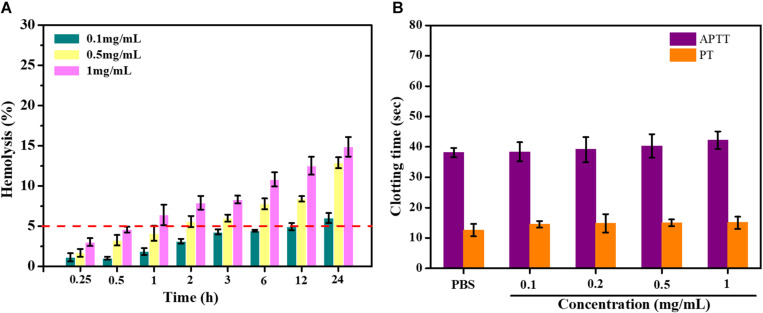
**(A)** Hemolysis assay of erythrocytes and blood of DSPE-PEG-RGD@ICG; **(B)** effect of DSPE-PEG-RGD@ICG on APTT and PT.

Hemolysis is the rupture of RBCs releasing their contents (hemoglobin, etc.) into the surroundings. Hemolytic activity of erythrocytes is an alternative and reliable way to assess the blood compatibility of drugs for intravenous administration. The American Society for Testing and Materials (ASTM F756) divided the materials into three categories: non-hemolytic (hemolysis: 0–2%), slightly hemolytic (hemolysis: 2–5%), and hemolytic (hemolysis: >5%). As shown in Figure 5A, the hemolysis was nearly 5% within a certain concentration range of DSPE-PEG-RGD@ICG. The hemolytic activity of DSPE-PEG-RGD@ICG showed dose-depended hemolysis. As the concentration increased, more erythrocytes ruptured. The results of the hemolysis assay indicated that the hemolytic toxicity of DSPE-PEG-RGD@ICG was permissible within its normal concentration range.

Blood clotting results from the plasma coagulation cascade, which is activated by platelets. APTT and PT represent the intrinsic and extrinsic pathways of coagulation time, respectively. The normal physiological levels for APTT and PT were 25.1∼36.5 s and 9.4∼12.5 s, respectively (Huang et al., 2016). The impact of micelles on coagulation factors was assessed in this section. After being treated with DSPE-PEG-RGD@ICG micelles in different concentrations, the coagulation time of the platelet-poor plasma (PPP) mixed with different reagents was tested. The results were shown in Figure 5B. The APTT and PT values of the PBS group were 38.10 ± 1.52 s and 12.60 ± 2.03 s. Meanwhile, DSPE-PEG-RGD@ICG had no significant effect on coagulation time for the two pathways from low concentration (0.1 mg/ml) to relatively high concentration (1 mg/ml). The APTT and PT value of DSPE-PEG-RGD@ICG in 1 mg/ml were 42.18 ± 2.93 s and 15.00 ± 2.00 s. Thus, at the experimental concentration, the APTT and PT values of DSPE-PEG-RGD@ICG were both in the normal range and did not initiate the coagulation pathway, showing a good blood biocompatibility.

### *In vivo* Acute Toxicity Test

**FIGURE 6:**
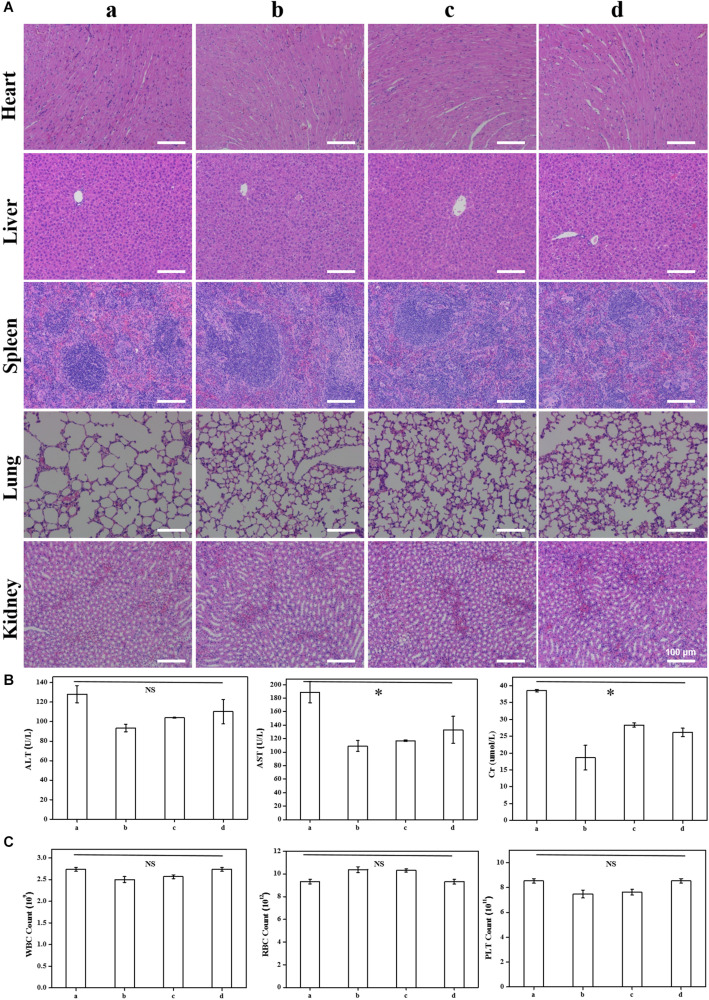
H&E staining of the main organs 7 days after intravenous injection **(A)**; blood parameters for evaluating effects on liver (ALT and AST) and kidney (Cr) function **(B)** and bone marrow hematopoietic system (RBC, WBC, and platelet) **(C)** in nude mice. Note that **(a–d)** stand for intravenous injection of DSPE-PEG-RGD@ICG with different doses: 0 mg/kg **(a)**, 1 mg/kg **(b)**, 2 mg/kg **(c)**, and 4 mg/kg **(d)**. ^∗^*P* < 0.05.

Based on the above research *in vitro*, we carried out the study of toxicity *in vivo*. Briefly, twelve healthy male nude mice were treated with different doses of DSPE-PEG-RGD@ICG. No mortality or other abnormal signs were observed throughout the entire 7-day study period. On the 8th day, blood samples and the major organs were harvested for further histopathological analysis. Figure 6A shoed the H&E staining images of the major organs of mice. There was no obvious influence on the tissue structure and morphology of the major organs in all groups. Blood chemistry parameters for evaluating liver (ALT and AST) and kidney (Cr) function were tested, which showed no significant changes (Figure 6B). Additionally, the whole blood cell counts also illustrated no significant difference in WBC, RBC, and platelet when compared to the PBS group (Figure 6C). Therefore, our results indicated that DSPE-PEG-RGD@ICG micelles are safe and promising materials for GC fluorescence imaging *in vivo*.

### *In vivo* Fluorescence Imaging and Distribution

**FIGURE 7:**
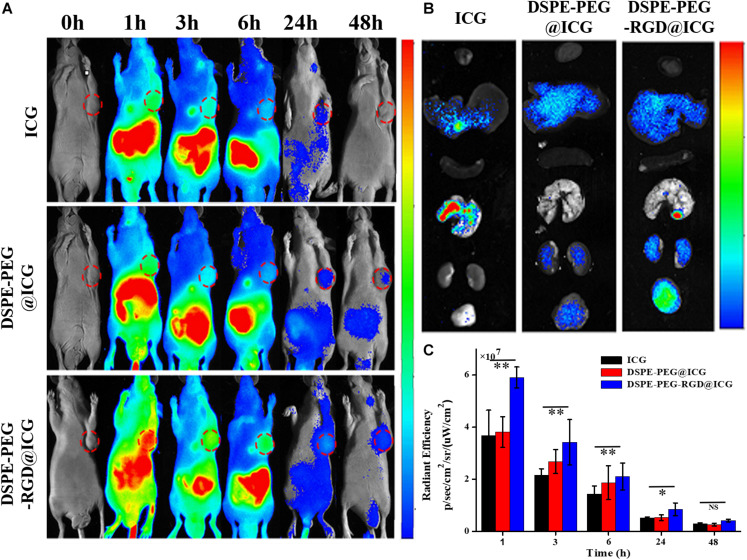
**(A)** Fluorescence signal of experimental mice at different times treated with free ICG, DSPE-PEG@ICG and DSPE-PEG-RGD@ICG, respectively. The red circle represents the location of the tumor. **(B)** *In vitro* fluorescence signal of tumors and organs of the mice were observed at 48 h after injection. **(C)** Changes of the fluorescence signal intensity at the tumor site. ^∗^*P* < 0.05 and ^∗∗^*P* < 0.01.

In order to verify the specific active targeting efficiency of DSPE-PEG-RGD@ICG toward GC, the fluorescence imaging of tumor-bearing mice treated with different reagents was observed by an optic imaging system. After the injection of free ICG or micelles with or without RGD via tail vein for 48 h, the real-time distribution and tumor accumulation of different fluorescence agents were shown in Figure 7A. Obviously, at 1 h post-injection, the fluorescence signal could be obtained all over the body with the help of blood circulation in the three groups, mainly of which was concentrated in the liver. However, the fluorescence accumulation of the tumor and the location and edge of the tumor could be observed in the DSPE-PEG-RGD@ICG group at that point, but not obvious in DSPE-PEG@ICG or ICG treated mice. The fluorescence intensity of the three groups decreased gradually with the extension of time (Figure 7C). After 24 h, there was only a small amount of fluorescence signal remaining in the gastrointestinal tract and tumor in the mice receiving free ICG. Previous studies indicated that ICG binds to plasma proteins such as albumin and rapidly metabolized from the liver when introduced into the blood circulation (Han et al., 2018). Our experimental results were consistent with this point. On the contrary, there still existed fluorescence in the mice injected with DSPE-PEG@ICG or DSPE-PEG-RGD@ICG. Moreover, the tumor fluorescence could still be observed in the two groups even after 48 h post-injection, indicating a prolonged circulation time. It might be explained that the encapsulation of ICG into micelles prevented ICG from binding to plasma proteins. Additionally, the fluorescence intensity of mice receiving DSPE-PEG-RGD@ICG was higher than that of mice receiving DSPE-PEG@ICG at indicated time points. Thus, the attachment of RGD polypeptide to micelles increased the accumulation of ICG in tumors via the interaction between RGD peptide and Integrin α_v_β_3_. After being imaged, all the tumor-bearing mice were immediately killed for isolating and visualizing the major organs and the tumors under the same conditions. As shown in Figure 7B, the *ex vivo* image conformed the obvious fluorescence accumulation in the tumors of the DSPE-PEG-RGD@ICG, but not the free ICG or DSPE-PEG@ICG. Interestingly, the fluorescence signals in the liver and spleen were also relatively high, which might be related to the natural phagocytosis of macrophages in these organs (Gref et al., 2012). So, how to minimize the nature internalization of micelles by the RES is a focus for the coming research. Thus, our results suggested that DSPE-PEG-RGD@ICG was an ideal fluorescence agent to higher accumulate in GC, and the EPR effect and RGD modification facilitated the targeting ability to tumors.

## Conclusion

In this study, we attached RGD polypeptide to DSPE-PEG-Mal through the Michael addition reaction, and successfully encapsulated ICG to construct the targeting fluorescent agent delivery micelles DSPE-PEG-RGD@ICG, in which RGD was used for active targeting to GC, and micelles were used to extend the circulation time *in vivo*. The particle size less than 200 nm was conducive to the passive targeting to tumors via the EPR effect. Meanwhile, the toxicity was systematically evaluated. Cytotoxicity and blood compatibility *in vitro* were consistent with the acute toxicity test *in vivo*, which revealed good biocompatibility at the experimental concentration. Further biodistribution and tumor targeting image showed an improved accumulation in tumors and longer circulation time when compared with free ICG or DSPE-PEG@ICG. In a word, this targeting delivery micelles possessed marked biocompatibility and improved targeting accumulation in tumors, thus providing a promising strategy to realize an early diagnosis and complete resection of GCs.

## Data Availability Statement

All datasets presented in this study are included in the article/Supplementary Material.

## Ethics Statement

The animal study was reviewed and approved by the Ethics Committee of the Institutional Animal Care and Use Subcommittee of the Third Hospital of Sun Yat-sen University.

## Author Contributions

BW designed the research. JS, XiZ, LF, TL, and DD performed the experiments. ZC, XuZ, and RL analyzed the data. JS and BW wrote the manuscript. All authors have read and approved the final submitted manuscript.

## Conflict of Interest

The authors declare that the research was conducted in the absence of any commercial or financial relationships that could be construed as a potential conflict of interest.
