# Supplementary material for: Targeting Fluorescence Imaging of RGD-Modified Indocyanine Green Micelles on Gastric Cancer
Source: Front Bioeng Biotechnol. 2020 Sep 25;8:575365. doi: 10.3389/fbioe.2020.575365 (PMC7546337; doi:10.3389/fbioe.2020.575365)
Supplement: Supplementary file 1 [file Image_1.pdf]

Targeting fluorescence imaging of RGD-modified indocyanine green micelles on gastric cancer

Jun Shao [a 1], Xiaoming Zheng [a, 1], Longbao Feng [b], Tianyun Lan [c], Dongbing Ding [a], Zikai Cai [a], Xudong Zhu [a], Rongpu Liang [a], Bo Wei [a *]

[a] Department of Gastrointestinal Surgery, the Third Affiliated Hospital of Sun Yat-sen University, Guangzhou 510630, China

[b] Department of Biomedical Engineering, Ji'nan University, Guangzhou 510630, China

[c] Central Laboratory, the Third Affiliated Hospital of Sun Yat-sen University, Guangzhou 510630, China

[1] These authors contributed equally to this work.

* Corresponding author: weibo3@mail.sysu.edu.cn

Telephone: +86-20-85252228

Figure Caption

Figure S1. ^{1}H NMR spectra of DSPE-PEG-Mal and DSPE-PEG-RGD.

Figure S2. The standard absorption curve of ICG in ultra-pure water.

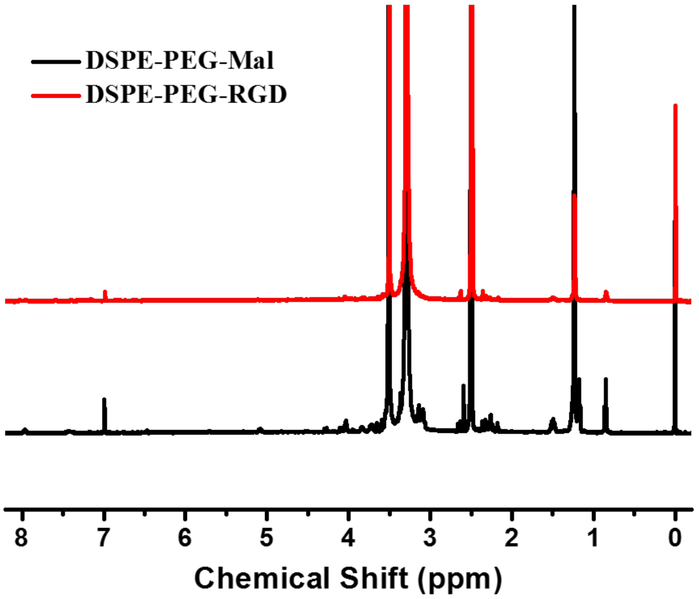

Figure S1

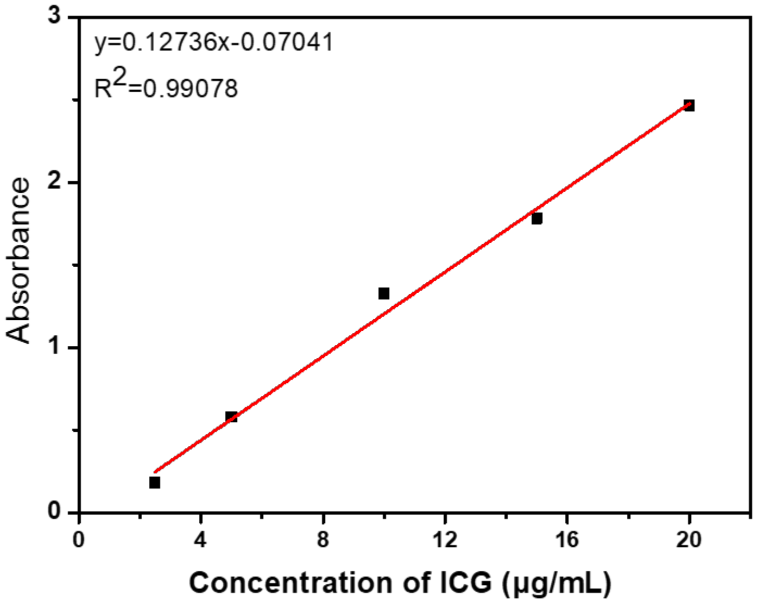

y=0.12736x-0.07041
R2=0.99078
Absorbance
Concentration of ICG (μg/mL)
0
1
2
3
0
4
8
12
16
20

Figure S2
